# High Levels of Epstein–Barr Virus Nuclear Antigen-1-Specific Antibodies and Infectious Mononucleosis Act Both Independently and Synergistically to Increase Multiple Sclerosis Risk

**DOI:** 10.3389/fneur.2019.01368

**Published:** 2020-01-24

**Authors:** Anna Karin Hedström, Jesse Huang, Angelica Michel, Julia Butt, Nicole Brenner, Jan Hillert, Tim Waterboer, Ingrid Kockum, Tomas Olsson, Lars Alfredsson

**Affiliations:** ^1^Institute of Environmental Medicine, Karolinska Institutet, Stockholm, Sweden; ^2^Department of Clinical Neuroscience, Karolinska Institutet, Stockholm, Sweden; ^3^Center for Molecular Medicine, Karolinska Institutet at Karolinska University Hospital, Stockholm, Sweden; ^4^Infections and Cancer Epidemiology, German Cancer Research Center, Heidelberg, Germany

**Keywords:** multiple sclerosis, Epstein–Barr virus infection, anti-EBNA-1 antibodies, infectious mononucleosis, human leukocyte antigen

## Abstract

**Objective:** Elevated levels of anti-EBNA-1 antibodies and infectious mononucleosis (IM) history have consistently been associated with multiple sclerosis (MS) risk. We aimed to study whether these aspects of Epstein–Barr virus (EBV) infection represent separate risk factors for MS and whether they both interact with MS-associated HLA genes in disease development.

**Methods:** Two Swedish-population-based case–control studies were used, comprising 5,316 cases and 5,431 matched controls. Subjects with different HLA alleles, EBNA-1, and IM status were compared regarding MS risk by calculating odds ratios (OR) with 95% confidence intervals (CI) employing logistic regression. Causal mediation analysis was used to assess to what extent the relationship between IM history and MS risk was mediated by high anti-EBNA-1 antibody levels and vice versa.

**Results:** The causal mediation analysis revealed that both aspects of EBV infection mainly act directly on MS risk. The direct effect of elevated anti-EBNA-1 antibody levels on MS risk, expressed on the OR scale, was 2.8 (95% CI 2.5–3.1), and the direct effect of IM history on MS risk was 1.7 (95% CI 1.5–2.0). A significant interaction between the two aspects of EBV infection was observed (RERI 1.2, 95% CI 0.3–2.0), accounting for about 50% of the total effect. Further, both aspects of EBV infection interacted with DRB1^*^15:01 and absence of A^*^02:01.

**Interpretation:** Elevated anti-EBNA-1 antibody levels and IM history are different risk factors for MS. The two aspects of EBV infection act synergistically to increase MS risk, indicating that they partly are involved in the same biological pathways.

## Introduction

Multiple sclerosis (MS) is an inflammatory demyelinating disease in which the etiology involves both genetic and environmental factors. The HLA class II allele DRB1^*^15:01 exerts the single strongest effect (1, 2), but several alleles within the HLA region have been shown to influence MS risk independently of DRB1^*^15:01 status, including A^*^02:01 which is negatively associated with MS (2, 3).

High levels of anti-EBNA-1 antibodies, which may be a marker of a deficient response to EBV infection, have consistently been associated with increased MS risk (4). Another consistent finding is the association between history of infectious mononucleosis (IM), indicative of post-childhood acquisition of EBV infection, and increased MS risk (5). However, it is unknown whether high anti-EBNA-1 antibody levels and IM history represent separate risk factors for MS.

Data from several studies suggest that the presence of DRB1^*^15:01 and high levels of anti-EBNA-1 antibody levels act synergistically to increase the risk of MS (6, 7). In the largest study on this topic, DRB1^*^15:01 carriers without the A^*^02:01 allele, with high levels of anti-EBNA-1 antibodies, had a 16-fold higher risk of MS than those who did not carry any of these factors (8). Similarly, an interaction between the same MS-associated HLA genes and IM history has been observed (9). No study has been powered enough to investigate the potential interaction between HLA genes and EBNA-1 status with regard to MS risk, taking IM history into consideration and vice versa.

Using two population-based case–control studies, we aimed to study whether an altered antibody response to EBNA-1 antigens reflects a higher prevalence of IM history among MS patients or whether elevated levels of anti-EBNA-1 antibodies and IM history represent separate risk factors. We also aimed to clarify what aspect or aspects of EBV infection interact with MS-associated HLA genes.

## Methods

This study is based on Epidemiological Investigation of Multiple Sclerosis (EIMS) and Genes and Environment in Multiple Sclerosis (GEMS), which are Swedish-population-based case–control studies, with a study base comprising the general population aged 16–70 years.

EIMS recruited incident cases of MS from neurology clinics between April 2005 and June 2015. Two controls per case were randomly selected from the national population register, frequency-matched for the cases' age in 5-year age strata, gender, and residential area.

GEMS identified prevalent cases, distinct from those in EIMS, from the Swedish National MS registry, and controls were randomly selected from the national population register, matched for age, gender, and residential area at the time of disease onset. The study participants were recruited between November 2009 and November 2011. All cases in both studies fulfilled the McDonald criteria (10). Ethical approval for both EIMS and GEMS was given by the Regional Ethical Review Board at Karolinska Institutet, and all participants provided informed consent.

### Data Collection and Exposure Information

Information regarding environmental exposures and lifestyle factors was collected by means of standardized questionnaires. The response rate was 93% for cases and 73% for controls in EIMS and 82% for cases and 66% for controls in GEMS. The subjects were asked about a history of IM with the answer alternatives: “yes,” “no,” or “do not know.” IM was recorded as either reported infection or no infection. Those who were unsure regarding a history of IM were excluded. All participants in both studies were asked to provide a blood sample, and those who did not were excluded. The number of subjects in each study is presented in Table 1.

**Table 1:** Number of cases and controls included in the study.

**Study**		**Included in the study**	**Data on HLA genotype and anti-EBNA-1 status**	**Data on infectious mononucleosis (dataset for analysis)**
EIMS	Cases	2,880	2,033	1,835
	Controls	6,122	2,458	2,270
GEMS	Cases	6,156	4,319	3,481
	Controls	5,408	3,770	3,161
Total	Cases	9,036	6,352	5,316
	Controls	11,530	6,228	5,431

### Genotyping and Measurement of Anti-EBNA-1 Antibody Levels

HLA-DRB1 and HLA-A alleles were determined at four-digit resolution. Genotyping was performed on the MS replication chip (11), which is based on an Illumina exome chip to which ~90,000 custom markers were added with extra-high density in the HLA region, and HLA alleles were then imputed with HLA^*^IMP:02 (12).

A multiplex serological assay using beads loaded with recombinant glutathione s-transferase fusion proteins was used for detection of IgG antibodies against the EBNA1 peptide segment (aa 385–420) (13, 14), which has been identified as the primary EBNA1 fragment associated with MS risk (9). Dual-laser flow-based detection was used to quantify the antibodies as units of median flourescence intensity. Anti-EBNA-1 antibody levels were dichotomized based on the median among controls, defining groups with high and low anti-EBNA-1 antibody levels. We also categorized anti-EBNA-1 antibody levels based on the 25th, 50th, and 75th percentiles among controls in order to perform a sensitivity analysis.

### Statistical Analysis

Subjects with different HLA alleles and EBNA-1 status (and IM status) were compared with regard to MS risk by calculating odds ratios (OR) with 95% confidence intervals (CI) using unconditional logistic regression models. Trend test for a dose–response relationship regarding anti-EBNA-1 antibody levels and risk of MS was performed by using a continuous variable for anti-EBNA-1 antibody levels in a logistic regression model.

We categorized subjects based on EBNA-1 and IM status and studied the influence of each factor in the absence of the other factor. When studying both factors simultaneously, causal mediation analysis was used to assess to what extent the relationship between past IM and MS risk was mediated by high anti-EBNA-1 antibody level and vice versa. The causal effects were estimated on the OR scale, and the CI values were calculated using the delta method (15). Further, a potential interaction on the additive scale was calculated between high EBNA-1 antibody levels and IM status, using the relative excess risk due to interaction (RERI) together with a 95% confidence interval.

Potential interactions between HLA alleles and both aspects of EBV infection were also calculated. We studied the total three-way interaction between HLA-DRB1^*^15:01, absence of HLA-A^*^02:01, and aspects of EBV infection (EBNA-1 status and IM, respectively) with regard to MS risk, comparing the joint effect of the three risk factors to the situation when each one acts separately. The total three-way interaction thus takes all two-way interactions and the three-way interaction into account. The interaction analysis on DRB1^*^15:01, absence of A^*^02:01, and EBNA-1 status was stratified by IM history and vice versa.

All analyses were adjusted for study, age, gender, residential area (county), ancestry, and the following alleles within the HLA region that have been shown to influence MS susceptibility independently of DRB1^*^15:01 status: DRB1^*^03:01, DRB1^*^13:03, DRB1^*^08:01, B^*^44:02, B^*^38:01, B^*^44:02, DQA1^*^01:01, DQB1^*^03:01, and DQB1^*^03:02. Homozygote correction was made for DRB1^*^15:01, DRB1^*^03:01, and A^*^02:01. Age was categorized into the following eight intervals: 16–19, 20–24, 25–29, 30–34, 35–39, 40–45, 45–49, and 50–70 years of age. Residential area assessment of ancestry was based on whether the subject was born in Sweden or not and whether either of the subject's parents had immigrated to Sweden. A subject who was born in Sweden and whose parents had not immigrated was classified as Swedish. The remaining subjects were classified as non-Swedish.

When appropriate we also adjusted for smoking, adolescent BMI, DRB1^*^15:01, and A^*^02:01.

The time of the initial appearance of MS symptoms was used as an estimate of the disease onset, and the year in which this occurred was defined as the index year. The corresponding controls were given the same index year. Smoking habits were only considered before and at the index year. Subjects were classified into never smokers, current smokers (who smoked at the index year), and past smokers (who had smoked prior to the index year). Adolescent BMI was calculated by dividing self-reported weight in kilograms at age 20 years by self-reported height in meters squared and dichotomized into BMI ≤ 25 or BMI > 25.

We additionally adjusted the analyses for passive smoking (yes or no), sun exposure habits, education, and socioeconomic index, but these variables only had minor influence on the results and were not retained in the final analyses. Based on three questions regarding exposure to ultraviolet radiation (UVR) where each answer alternative was given a number ranging from 1 (the lowest exposure) to 4 (the highest exposure), we constructed an index by adding the numbers together and thus acquired a value between 3 and 12. Educational level was categorized into no post-secondary education, post-secondary education without university degree, or university degree. The last occupation during the year before the index year was used as a marker for socioeconomic class which was categorized into the following strata: 1, workers in goods production; 2, workers in service production; 3, employees at lower and intermediate levels; 4, employees at higher levels, executives, and university graduates; and 5, others such as pensioners, students, and unemployed.

In order to evaluate the presence of bias due to the exclusion of cases and controls with missing data on IM history, we performed several sensitivity analyses. The distribution of anti-EBNA-1 antibody levels among cases with unknown IM history was compared with that of cases that remained in the final analyses. A corresponding analysis was conducted among the controls. A sensitivity analysis was conducted in which the influence of IM on MS risk was calculated across categories of anti-EBNA-1 antibody levels, based on the 25th, 50th, and 75th percentiles among controls.

The interaction between DRB1^*^15:01, absence of A^*^02:01, and elevated anti-EBNA-1 antibody levels was performed, including also those with missing data on IM history after imputing missing data using the multiple imputation chained equation procedure (16). We also performed fictive analyses regarding the interaction between DRB1^*^15:01, absence of A^*^02:01, and past IM, one in which unknown IM history was replaced by a positive answer and one in which unknown IM history was replaced by a negative answer. Finally, all analyses were additionally performed restricted to include subjects of Swedish origin. All analyses were conducted using Statistical Analysis System (SAS) version 9.4.

## Results

**Figure 1:**
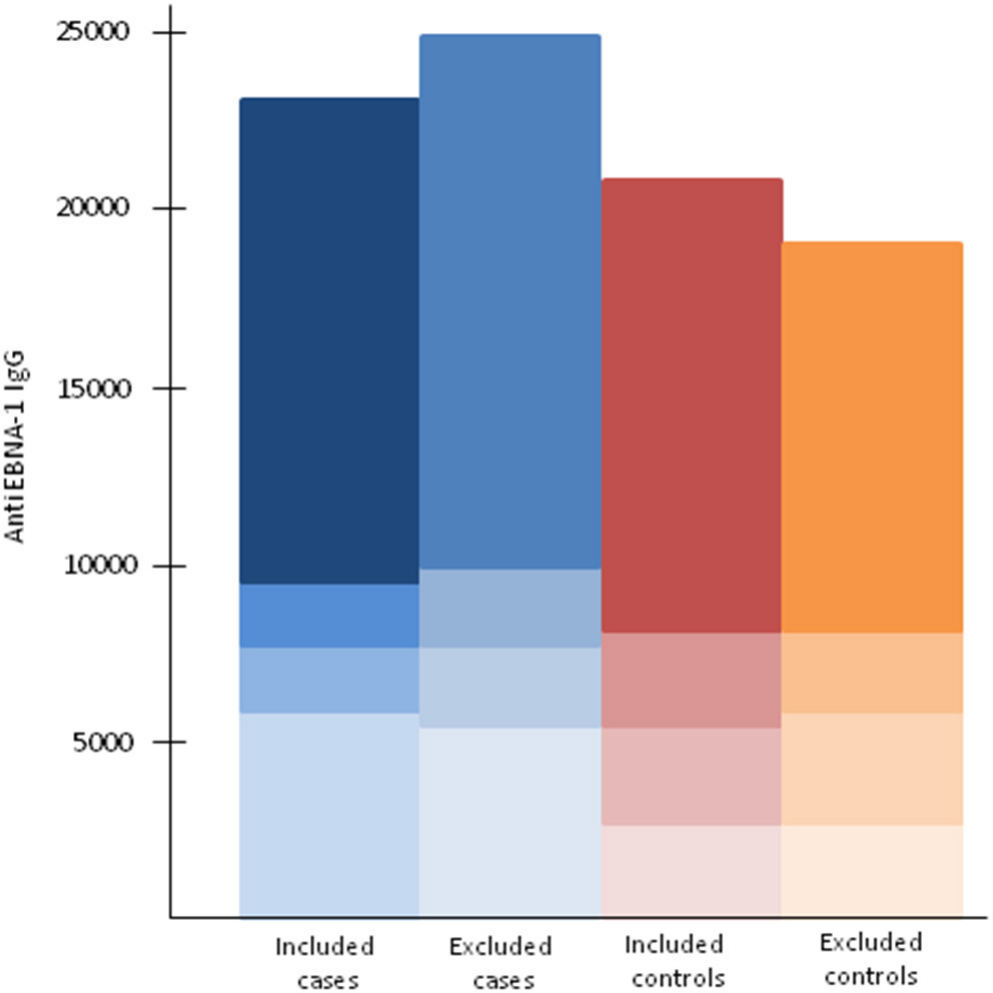
Anti-EBNA-1 antibody levels among included and excluded cases and controls by the 25th, 50th, and 75th percentiles among controls.

Our analyses regarding MS-associated HLA genes and aspects of EBV infection included 5,316 cases and 5,431 controls (Table 1). The characteristics of cases and controls, by different combinations of anti-EBNA-1 and IM status, are presented in Table 2. Participants who were excluded due to unknown IM history did not differ with regard to anti-EBNA-1 antibody levels or frequency of DRB1^*^15:01 or A^*^02:01 status compared to those with known IM history (Table 3; Figure 1).

**Table 2:** Characteristics of cases and controls by different combinations of anti-EBNA-1 and IM status.

	**Low anti-EBNA-1 antibody levels IM** **=** **0**		**Low anti-EBNA-1 antibody levels IM** **=** **1**		**High EBNA-1 antibody levels IM** **=** **0**		**High anti-EBNA-1 antibody levels IM** **=** **1**	
	**Cases**	**Controls**	**Cases**	**Controls**	**Cases**	**Controls**	**Cases**	**Controls**
Women, *n* (%)	793 (74)	1,876 (77)	134 (80)	178 (81)	2,501 (73)	1,915 (77)	482 (75)	230 (82)
Men, *n* (%)	274 (26)	569 (23)	34 (20)	42 (19)	939 (27)	569 (23)	159 (25)	52 (18)
Swedish, *n* (%)	841 (78)	1,951 (80)	141 (84)	179 (81)	2,813 (82)	2,004 (81)	540 (84)	235 (83)
Median anti-EBNA-1 antibody levels	3,597	2,573	3,938	2,837	8,549	7,938	8,648	7,730
**Smoking status**								
Never smoking, *n* (%)	479 (45)	1,326 (54)	90 (54)	130 (59)	1,510 (44)	1,299 (52)	290 (45)	159 (56)
Current smoking, *n* (%)	360 (34)	671 (27)	45 (28)	51 (23)	1,264 (37)	700 (28)	224 (35)	59 (21)
Past smoking, *n* (%)	228 (21)	448 (18)	33 (20)	39 (18)	666 (19)	485 (20)	127 (20)	64 (23)
Mean adolescent BMI (SD)	21.9 (3.4)	21.7 (3.6)	22.0 (3.3)	21.5 (2.7)	21.9 (4.5)	21.8 (4.6)	22.5 (4.8)	21.5 (2.5)
DRB1*15:01 status								
Negative, *n* (%)	579 (54)	1,850 (76)	89 (53)	174 (79)	1,323 (38)	1,665 (67)	244 (38)	188 (67)
Heterozygotes, *n* (%)	413 (39)	546 (22)	68 (40)	42 (19)	1,729 (50)	734 (30)	321 (50)	86 (31)
Homozygotes, *n* (%)	75 (7.0)	49 (2.0)	11 (6.6)	4 (1.8)	388 (11)	85 (3.4)	76 (12)	8 (2.8)
A*02:01 status								
Negative, *n* (%)	550 (52)	1,083 (44)	104 (62)	97 (44)	1,968 (57)	1,099 (44)	421 (66)	139 (49)
Heterozygotes, *n* (%)	416 (39)	1,060 (43)	49 (29)	104 (47)	1,257 (37)	1,108 (45)	184 (29)	114 (40)
Homozygotes, *n* (%)	101 (9.5)	302 (12)	15 (8.9)	19 (8.6)	215 (6.3)	277 (11)	36 (5.6)	29 (10)
Total	1,067	2,445	168	220	3,440	2,484	641	282

**Table 3:** Differences between cases and controls who did or did not provide information regarding IM history.

	**Cases**			**Controls**		
	**IM history**		***P* value for difference between groups**	**IM history**		***P* value for difference between groups**
	**Known**	**Unknown**		**Known**	**Unknown**	
Women, *n* (%)	3,910 (74)	686 (67)	< 0.0001	4,199 (77)	540 (69)	< 0.0001
Swedish, *n* (%)	4,335 (82)	781 (76)	0.0002	4,369 (80)	572 (73)	< 0.0001
Median anti-EBNA-1 antibody levels	7,802	7,566	0.48	5,603	5,703	0.72
**Smoking status**						
Never, *n* (%)	2,369 (45)	405 (40)		2,914 (54)	384 (49)	
Current, *n* (%)	1,893 (36)	432 (42)		1,481 (27)	220 (28)	
Past, *n* (%)	1,054 (20)	187 (18)	0.08	1,036 (19)	184 (23)	0.003
Mean adolescent BMI (SD)	22.0 (4.3)	22.2 (6.1)	0.47	21.7 (4.0)	22.1 (6.2)	0.11
DRB1*15:01 status						
Negative, *n* (%)	2,235 (42)	451 (44)		3,877 (71)	590 (75)	
Heterozygotes, *n* (%)	2,531 (48)	476 (46)		1,408 (26)	176 (22)	
Homozygotes, *n* (%)	550 (10)	97 (9.5)	0.20	146 (2.7)	22 (2.8)	0.05
A*02:01 status						
Negative, *n* (%)	3,043 (57)	578 (56)		2,418 (45)	353 (45)	
Heterozygotes, *n* (%)	1,906 (36)	376 (37)		2,386 (44)	342 (44)	
Homozygotes, *n* (%)	367 (6.9)	70 (6.8)	0.68	627 (12)	93 (12)	0.96

Overall, elevated anti-EBNA-1 antibody levels increased MS risk by 3-fold (adjusted OR 3.1, 95% CI 2.9–3.4). The risk of MS increased with increasing anti-EBNA-1 antibody levels (*p* for trend <0.0001). There was only a weak correlation between anti-EBNA-1 antibody levels and IM (*r* = 0.02, *p* = 0.07 among controls and *r* = 0.03, *p* = 0.01 among cases).

Overall, IM history increased the risk of MS by 70% (adjusted OR 1.7, 95% CI 1.5–1.9). The influence of IM history on MS risk was significant across all quartiles of anti-EBNA-1 antibody levels (Table 4).

**Table 4:** OR with 95% CI of developing MS for subjects with a history of IM by categories of anti-EBNA-1 antibody levels based on quantiles among controls.

**Anti-EBNA1 antibody levels (quantiles among controls)**	**ca/co^**a**^**	**OR (95% CI)^**b**^**	**OR (95% CI)^**c**^**
<2,661 (<25)	48/102	1.6 (1.1–2.3)	1.6 (1.1–2.3)
2,661–5,602 (25–50)	122/124	1.7 (1.3–2.2)	1.8 (1.4–2.4)
5,603–7,951 (50–75)	227/148	1.4 (1.1–1.7)	1.4 (1.1–1.7)
7,952– (75–)	412/127	1.9 (1.6–2.4)	1.9 (1.6–2.4)

a *Number of exposed cases and controls*.

b *Adjusted for age, gender, residential area, study, and ancestry*.

c Adjusted for age, gender, residential area, study, ancestry, smoking, adolescent BMI, DRB1*03:01, DRB1*13:03, DRB1*08:01, B*44:02, B*38:01, B*55:01, DQA1*01:01, DQB1*03:01, and DQB1*03:02.

### Mediation Analysis

The total effect of elevated anti-EBNA-1 antibody levels on MS risk expressed as OR was 2.8 (95% CI 2.6–3.1). The direct effect was 2.8 (95% CI 2.5–3.1), and the indirect effect, mediated by IM, was 1.03 (0.99–1.02). Thus, the mediating effects were very small.

The total effect of IM history on MS risk was 1.8 (95% CI 1.5–2.0). The direct effect was 1.7 (95% CI 1.5–2.0), whereas the indirect effect, mediated by high anti-EBNA-1 antibody levels, was negligible (OR 1.01, 95% CI 0.98–1.18).

### Interaction Between Anti-EBNA-1 Status and IM

When subjects were categorized based on EBNA-1 status and IM history, each factor increased MS risk in the absence of the other; high anti-EBNA-1 antibody levels conferred a 3-fold increased risk of disease (adjusted OR 3.1, 95% CI 2.8–3.3), whereas IM history increased MS risk by 50% (adjusted OR 1.5, 95% CI 1.4–1.7). Compared with subjects with low anti-EBNA-1 antibody levels without IM history, there was a significant interaction between high anti-EBNA-1 antibody levels and IM history among subjects exposed to both factors (RERI 1.2, 95% CI 0.3–2.0) (Table 5). Interaction between high anti-EBNA-1 antibody levels and IM history accounted for ~50% of the total effect.

**Table 5:** OR with 95% CI of developing MS for subjects with different combinations of anti-EBNA-1 status and IM history compared to subjects with low anti-EBNA-1 antibody levels without IM history (relative access proportion due to interaction, RERI).

**Anti-EBNA-1 antibody levels**	**IM history**	**ca/co^**a**^**	**OR (95% CI)^**b**^**	**OR (95% CI)^**c**^**
Low	–	1,067/2,445	1.0 (reference)	1.0 (reference)
Low	+	168/220	1.8 (1.4–2.2)	1.8 (1.5–2.3)
High	–	3,440/2,484	3.2 (2.9–3.5)	3.2 (2.9–3.5)
High	+	641/282	5.2 (4.4–6.1)	5.2 (4.4–6.1)
				RERI 1.2 (0.3–2.0)

a *Number of exposed cases and controls*.

b *Adjusted for age, gender, residential area, study, and ancestry*.

c Adjusted for age, gender, residential area, study, ancestry, smoking, adolescent BMI, DRB1*15:01, DRB1*03:01, DRB1*13:03, DRB1*08:01, A*02:01, B*44:02, B*38:01, B*55:01, DQA1*01:01, DQB1*03:01, and DQB1*03:02.

### Interaction Between HLA-DRB1^*^15:01 and HLA-A^*^02:01 Alleles and EBNA-1 and IM, Respectively

Among cases and controls, the DRB1^*^15:01 allele was significantly more common among those with high than among those with low anti-EBNA-1 antibody levels (*p* < 0.0001 for both cases and controls, respectively), whereas DRB1^*^15:01 frequency did not differ by IM status. Among cases, A^*^02:01 was significantly less common among those who reported a history of IM (*p* < 0.0001) regardless of EBNA-1 status (Table 2).

There was a three-way interaction between DRB1^*^15:01, absence of A^*^02:01, and EBNA-1 status regardless of IM status (Table 6). The combination of the genetic risk factors among subjects with low anti-EBNA-1 antibody levels increased the risk of MS with an OR of 4.9 (95% CI 3.9–6.1), whereas high EBNA-1 antibody levels rendered an OR of 2.3 (95% CI 1.9–2.7) among those without the genetic risk factors. However, subjects with high anti-EBNA-1 antibody levels with the genetic risk factors had a 17-fold increased risk of MS (OR 16.8, 95% CI 14.0–20.2). The formal evaluation of the interaction between DRB1^*^15:01, absence of A^*^02:01, and EBNA-1 status rendered a RERI of 11.6 (95% CI 9.1–14.0).

**Table 6:** OR with 95% CI of developing MS for subjects with different combinations of DRB1*1501, A*0201, and EBNA-1 status compared to subjects with low anti-EBNA-1 antibody levels without the genetic risk factors, overall and stratified by IM status (relative access proportion due to interaction, RERI).

			**Total**			**History of IM**		**No history of IM**	
DRB1*15:01	A*02:01	**Anti-EBNA-1 antibody levels**	**ca/co^**a**^**	**OR (95% CI)^**b**^**	**OR (95% CI)^**c**^**	**ca/co^**a**^**	**OR (95% CI)^**d**^**	**ca/co^**a**^**	**OR (95% CI)^**d**^**
–	+	Low	293/1,111	1.0 (reference)	1.0 (reference)	29/96	1.0 (reference)	264/1015	1.0 (reference)
–	–	Low	375/913	1.6 (1.3–1.9)	1.6 (1.3–1.9)	60/78	2.3 (1.3–4.1)	315/835	1.5 (1.2–1.8)
+	+	Low	288/374	3.4 (2.8–4.2)	3.4 (2.8–4.2)	35/27	4.4 (2.2–8.7)	253/347	3.3 (2.7–4.1)
+	–	Low	279/267	4.9 (3.9–6.1)	4.9 (3.9–6.1)	44/19	8.2 (4.0–16.8)	235/248	4.6 (3.6–5.8)
–	+	High	592/1012	2.3 (1.9–2.7)	2.3 (1.9–2.7)	77/96	2.5 (1.5–4.2)	515/916	2.2 (1.9–2.7)
–	–	High	975/841	4.5 (3.8–5.3)	4.5 (3.8–5.3)	167/92	5.2 (3.2–8.7)	808/749	4.3 (3.6–5.1)
+	+	High	1,100/516	9.4 (7.9–11.2)	9.4 (7.9–11.2)	143/47	9.8 (5.6–17.2)	957/469	9.4 (7.8–11.3)
+	–	High	1,414/397	16.8 (14.0–20.2)	16.8 (14.0–20.2)	254/47	18.9 (10.8–32.9)	1,160/350	16.2 (13.4–19.7)
					RERI 11.6 (9.1–14.0)		RERI 11.7 (3.6–19.7)		RERI 11.2 (8.6–13.7)

a *Number of exposed cases and controls*.

b *Adjusted for age, gender, residential area, study, and ancestry*.

c Adjusted for age, gender, residential area, study, ancestry, infectious mononucleosis, smoking, adolescent body mass index, DRB1*03:01, DRB1*13:03, DRB1*08:01, B*44:02, B*38:01, B*55:01, DQA1*01:01, DQB1*03:01, and DQB1*03:02.

d Adjusted for age, gender, residential area, study, ancestry, smoking, adolescent body mass index, DRB1*03:01, DRB1*13:03, DRB1*08:01, B*44:02, B*38:01, B*55:01, DQA1*01:01, DQB1*03:01, and DQB1*03:02.

Similarly, there was a three-way interaction between DRB1^*^15:01, absence of A^*^02:01, and IM regardless of anti-EBNA-1 status (Table 7). A history of IM among those without the genetic risk factors increased the risk of MS with an OR of 1.5 (95% CI 1.1–1.9). Compared to subjects with none of the three risk factors in question, those exposed to both genetic risk factors had an OR of 7.0 (95% CI 6.1–8.0), whereas those exposed to all three risk factors had a 14-fold increased risk of developing MS (OR 14.1, 95% CI 10.6–18.9). Evaluating the total interaction between the three risk factors rendered a RERI of 8.7 (95% CI 4.7–12.6).

**Table 7:** OR with 95% CI of developing MS for subjects with different combinations of DRB1^*^15:01, A^*^02:01, and IM history compared to subjects with no IM history and without the genetic risk factors, overall and stratified by anti-EBNA-1 status (relative access proportion due to interaction, RERI).

			**Total**			**High anti-EBNA-1 antibody levels**		**Low anti-EBNA-1 antibody levels**	
DRB1*15:01	A*02:01	**IM history**	**ca/co^**a**^**	**OR (95% CI)^**b**^**	**OR (95% CI)^**c**^**	**ca/co^**a**^**	**OR (95% CI)^**d**^**	**ca/co^**a**^**	**OR (95% CI)^**d**^**
–	+	–	779/1,931	1.0 (reference)	1.0 (reference)	515/916	1.0 (reference)	264/1,015	1.0 (reference)
–	–	–	1,123/1,584	1.8 (1.6–2.0)	1.8 (1.6–2.0)	808/749	1.9 (1.7–2.2)	315/835	1.5 (1.2–1.8)
+	+	–	1,210/816	3.7 (3.3–4.2)	4.2 (3.7–4.8)	957/469	4.1 (3.5–4.8)	253/347	3.3 (2.7–4.2)
+	–	–	1,395/598	5.9 (5.2–6.7)	7.0 (6.1–8.0)	1,160/350	7.1 (6.0–8.4)	235/248	4.6 (3.6–5.9)
–	+	+	106/192	1.4 (1.1–1.8)	1.5 (1.1–1.9)	77/96	1.5 (1.1–2.1)	29/96	1.3 (0.8–2.1)
–	–	+	227/170	3.3 (2.7–4.1)	3.4 (2.7–4.2)	167/92	3.2 (2.4–4.2)	60/78	3.0 (2.1–4.4)
+	+	+	178/74	6.0 (4.5–8.1)	6.8 (5.1–9.1)	143/47	6.1 (4.2–8.7)	35/27	6.3 (3.7–10.9)
+	–	+	298/66	11.4 (8.6–15.1)	14.1 (10.6–18.9)	254/47	11.9 (8.5–16.8)	44/19	11.9 (6.7–21.2)
					RERI 8.7 (4.7–12.6)		RERI 6.4 (2.5–10.3)		RERI 7.8 (1.0–14.6)

a *Number of exposed cases and controls*.

b *Adjusted for age, gender, residential area, study, and ancestry*.

c Adjusted for age, gender, residential area, study, ancestry, anti-EBNA1 status, smoking, adolescent body mass index, DRB1^*^03:01, DRB1^*^13:03, DRB1^*^08:01, B^*^44:02, B^*^38:01, B^*^55:01, DQA1^*^01:01, DQB1^*^03:01, and DQB1^*^03:02.

d Adjusted for age, gender, residential area, study, ancestry, smoking, adolescent body mass index, DRB1*03:01, DRB1*13:03, DRB1*08:01, B*44:02, B*38:01, B*55:01, DQA1*01:01, DQB1*03:01, and DQB1*03:02.

**Figure 2:**
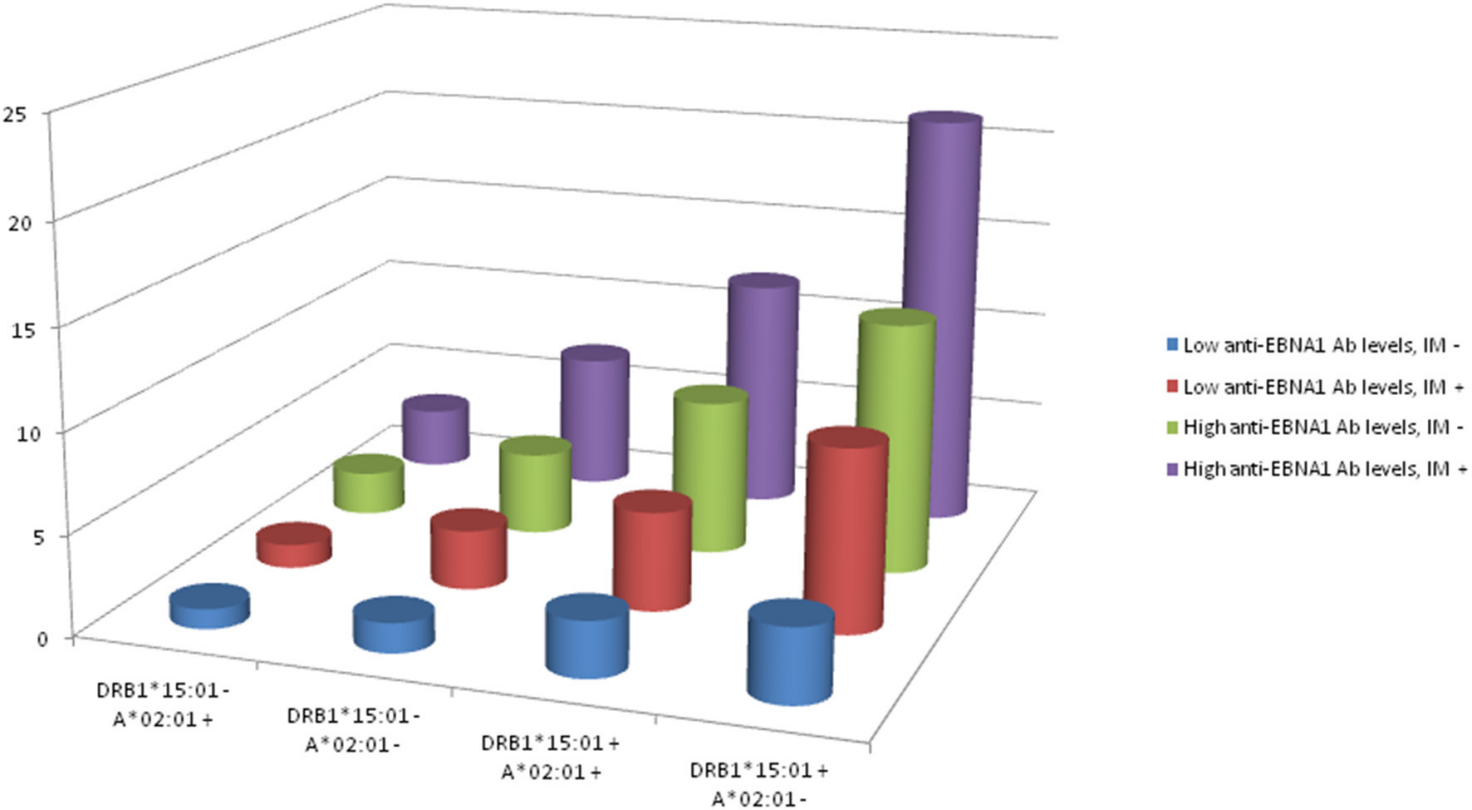
OR of developing MS among subjects with different combinations of DRB1*15:01, A*02:01, EBNA1, and IM status.

The interactions between DRB1^*^15:01, absence of A^*^02:01, anti-EBNA-1, and IM status are illustrated in Figure 2. The figure is based on data from Table 8 which present the OR of MS among subjects with different combinations of DRB1^*^15:01, A^*^02:01, EBNA-1, and IM status. Compared to subjects with low anti-EBNA-1 antibody levels, no IM history, and without the genetic risk factors, the OR was 27.1 (95% CI 19.1–38.5) among subjects with high anti-EBNA-1 antibody levels and IM history carrying both genetic risk factors.

**Table 8:** OR with 95% CI of developing MS among subjects with different combinations of DRB1*15:01, A*02:01, anti-EBNA-1, and IM status.

DRB1*15:01	A*02:01	**Anti-EBNA-1 antibody levels**	**IM**	**ca/co^**a**^**	**OR (95% CI)^**b**^**	**OR (95% CI)^**c**^**
-	+	Low	–	264/1,015	1.0 (reference)	1.0 (reference)
–	–	Low	–	315/835	1.5 (1.2–1.8)	1.5 (1.3–1.8)
+	+	Low	–	253/347	2.8 (2.3–3.5)	3.3 (2.7–4.2)
+	–	Low	–	235/248	3.7 (2.9–4.6)	4.5 (3.6–5.7)
–	+	Low	+	29/96	1.2 (0.8–1.8)	1.3 (0.8–2.1)
–	–	Low	+	60/78	3.0 (2.1–4.3)	3.1 (2.1–4.5)
+	+	Low	+	35/27	5.0 (3.0–8.4)	6.1 (3.6–10.4)
+	–	Low	+	44/19	9.2 (5.3–16.0)	11.5 (6.5–20.4)
–	+	High	–	515/916	2.2 (1.8–2.6)	2.2 (1.9–2.7)
–	–	High	–	808/749	4.2 (3.5–4.9)	4.3 (3.6–5.1)
+	+	High	–	957/469	7.9 (6.6–9.4)	9.2 (7.7–11.1)
+	–	High	–	1,160/350	12.8 (10.7–15.3)	16.0 (13.2–19.4)
–	+	High	+	77/96	3.1 (2.2–4.3)	3.3 (2.4–4.6)
–	–	High	+	167/92	6.9 (5.2–9.3)	7.1 (5.3–9.6)
+	+	High	+	143/47	11.8 (8.3–16.9)	13.6 (9.4–19.6)
+	–	High	+	254/47	21.3 (15.2–30.0)	27.1 (19.1–38.5)

a *Number of exposed cases and controls*.

b *Adjusted for age, gender, residential area, study, and ancestry*.

c Adjusted for age, gender, residential area, study, ancestry, smoking, adolescent BMI, DRB1*03:01, DRB1*13:03, DRB1*08:01, B*44:02, B*38:01, B*55:01, DQA1*01:01, DQB1*03:01, and DQB1*03:02.

We observed no suggestion of bias from the sensitivity analyses. The distribution of anti-EBNA-1 antibody levels was virtually the same among cases and controls with and without data on IM history. The results remained essentially unchanged after carrying out the analyses on the multiple imputed data, i.e., when subjects with missing data on IM history were included in the interaction analysis between DRB1^*^15:01, absence of A^*^02:01, and elevated anti-EBNA-1 antibody levels.

In the fictive analyses of DRB1^*^15:01, absence of A^*^02:01, and past IM, the interaction between the three risk factors remained significant when missing IM history was replaced by either a negative answer (RERI 7.8, 95% CI 4.2–11.5) or a positive answer (RERI 6.5, 95% CI 4.3–8.2). The results also remained similar when the analyses were further stratified by EBNA-1 status (data not shown). All main findings remained similar when the analyses were restricted to include subjects of Swedish ancestry (data not shown).

## Discussion

High anti-EBNA-l antibody levels and IM history seem to be different risk factors for MS. A significant interaction between the two aspects of EBV infection was observed, accounting for about 50% of the total effect, indicating that they are involved in the same biological pathways.

Cell-mediated immune processes play a pivotal role in controlling the number of EBV-infected B cells, and high anti-EBNA-1 antibody levels may reflect a deficient control of the EBV infection, increasing the tendency toward autoimmunity. How past IM contributes to further increase MS risk is not clear. Both individuals with subclinical infection and IM display similarly high levels of viral load in the blood, and the fraction of EBV-infected memory B cells following subclinical infection is similar to that observed in IM (17, 18). However, subclinical infection does not evoke a massive peripheral CD8+ T cell response, whereas the symptoms of IM are caused by the patient mounting an exaggerated cellular immune response (18). The outcome of primary EBV infection may be influenced by host genetics (19–21), an age-related impaired CD8+ T cell function (22–24), or changes in the CD8+ T cell repertoire in response to prior infections (25). However, the large expansions of T cells during IM may not be associated with a more efficient control of the primary infection, and it is possible that the exaggerated immune response in IM reflects another mechanism by which autoimmunity is increased.

Both high anti-EBNA-1 IgG and past IM interact with the same MS-associated HLA risk genes (9). Our result of an association between DRB1^*^15:01 and EBNA-1 status suggests that DRB1^*^15:01 affects the humoral response to EBV and is in accordance with previous findings of higher EBNA-1 titers among DRB1^*^15:01-positive individuals (21). To some extent, DRB1^*^15:01 may influence the risk of MS by preventing an effective immune response to EBV, leading to EBV accumulation in B cells. It has been proposed that EBV-infected autoaggressive B cells migrate to the organ containing the self-antigen that they recognize, further allowing the infiltration of CD4+ T cells (26). In genetically susceptible individuals, cross-reactivity between EBV and CNS antigens may induce an autoaggressive T cell response and subsequently lead to MS (27, 28).

HLA class I genes are also involved in controlling EBV infection. Genetic differences in the class I locus have been shown to influence both the outcome of the primary EBV infection and the viral persistence (20, 21). In the present study, the frequency of A^*^02:01 was significantly lower among cases with past IM compared to both cases without IM history and controls. Expression of A^*^02:01 molecules has been suggested to increase the negative selection of CNS autoreactive T cells or modulate their autoreactivity (29). Absence of A^*^02:01 may result in autoreactive T cells persisting and launching an immune response against the self-antigen. DRB1^*^15:01 and absence of A^*^02:01 may thus influence and promote each other, accelerating the progression of MS.

A number of vaccine strategies against EBV have been evaluated in clinical trials. None of them induced immunity that protected from infection, but some of them lowered the rate of IM (30). Whether the viral load in the blood will be lower among vaccinated people who become infected with EBV is uncertain. Further insight into the immune mechanisms that are critical to prevent or control EBV infection is needed. However, the long-term consequences of a vaccine against a virus that has co-evolved with humans for millions of years are unknown (31).

Both EIMS and GEMS are case–control studies in which personal information and information on exposures and lifestyle factors were collected retrospectively. There is a potential for recall bias, especially in GEMS who used prevalent cases, but the magnitude of memory errors is probably similar among cases and controls. There is also a risk of misclassification of reported IM among cases and controls since not all IM cases are caused by EBV.

A potential selection bias may arise during the recruitment process of cases and controls. Considering the structure of the Swedish healthcare system, which provides equal free-of-charge access to medical services for all citizen, MS cases are referred to neurological units, making them eligible to be part of the studies. In both studies, the problem of selection bias was minimized by the population-based design, and even though there was a relatively high proportion of non-responders among the controls, this bias is probably modest because the prevalence of lifestyle factors, such as smoking and alcohol consumption, among the controls was consistent with that of the general population in similar ages^1^. Male participants and participants of non-Swedish origin were less prone to provide information regarding IM history. However, there were no significant differences with regard to sex or ancestry between cases and controls who did not provide information on IM history. Furthermore, there were no significant differences with respect to age, gender, or smoking habits between those who provided a blood sample and those who did not, indicating that selection bias did not take place in this step. We consider it unlikely that our findings would be affected by bias to a large extent, especially since such a bias would then depend both on HLA genotype and anti-EBNA-1 antibody reactivity. We observed no suggestion of bias from the sensitivity analyses, but we cannot completely rule out a minor degree of bias in the estimated associations due to recall bias, exposure misclassification, or residual confounding.

Anti-EBNA-1 antibody levels were measured after MS onset, and these levels were assumed to reflect levels before disease onset. This assumption is supported by findings that elevations in anti-EBNA-1 antibody levels become increased between 15 and 20 years before the first symptoms of MS and thereafter remain constant over time (32).

In conclusion, elevated anti-EBNA1 antibody levels and IM history seem to be different risk factors for MS. The two aspects of EBV infection act synergistically to increase MS risk, indicating that they are involved in the same biological pathways. Both aspects of EBV infection interact with the same MS-associated HLA alleles with regard to MS risk.

## Data Availability Statement

Anonymized data will be shared by request from any qualified investigator that wants to analyse questions that are related to the published article.

## Ethics Statement

The studies involving human participants were reviewed and approved by Regional Ethical Review Board at Karolinska Institutet. Written informed consent from the participants' legal guardian/next of kin was not required to participate in this study in accordance with the national legislation and the institutional requirements.

## Author Contributions

AH was involved in study concept and design, statistical analysis, and drafting of the manuscript. JHu, AM, JB, NB, TW, and IK contributed in data extraction, interpretation of data, and critical revision of the manuscript. JHi, TO, and LA were engaged in study concept and design, interpretation of data, and critical revision of the manuscript.

### Conflict of Interest

IK received speaker's fees from Merck-Serono, and is involved in a project sponsored by Biogen. JHi received honoraria for serving on advisory boards for Biogen and Novartis and speaker's fees from Biogen, Merck-Serono, Bayer-Schering, Teva, and Sanofi-Aventis. He has served as principal investigator for projects sponsored by, or received unrestricted research support from, Biogen, Merck-Serono, TEVA, Novartis and Bayer-Schering. TO served on scientific advisory boards and received speaker honoraria Novartis, Merck-Serono, Biogen Idec, TEVA and Genzyme; served as Co-editor of Current Opinion in Immunology; received from Novartis and Biogen; and receives research support from Novartis, Genzyme, Biogen Idec, the Swedish Research Council (07488), EU fp7 Neurinox, and CombiMS, and the Swedish Brain Foundation. LA received research support from the Swedish Medical Research Council (Dnr 2016-02349), the Swedish Council for Health, Working Life and Welfare (Dnr 2015-00195) and the Swedish Brain Foundation; has received speaker honoraria from Biogen Idec and TEVA. The remaining authors declare that the research was conducted in the absence of any commercial or financial relationships that could be construed as a potential conflict of interest.

## Footnotes

^1^Internet Based Information. Available online at: http://www.scb.se (accessed April 16, 2019).
